# The Effects of Modest Alcohol Consumption on Non-alcoholic Fatty Liver Disease: A Systematic Review and Meta-Analysis

**DOI:** 10.3389/fmed.2021.744713

**Published:** 2021-08-27

**Authors:** Wasit Wongtrakul, Sorachat Niltwat, Phunchai Charatcharoenwitthaya

**Affiliations:** ^1^Division of Gastroenterology, Department of Medicine, Faculty of Medicine Siriraj Hospital, Mahidol University, Bangkok, Thailand; ^2^Department of Research and Development, Faculty of Medicine Siriraj Hospital, Mahidol University, Bangkok, Thailand; ^3^Division of Gastroenterology, Department of Medicine, Panyananthaphikkhu Chonprathan Medical Center, Srinakharinwirot University, Nonthaburi, Thailand

**Keywords:** non-alcoholic fatty liver disease, modest alcohol, histology, hepatocellular carcinoma, mortality, NAFLD, meta-analysis

## Abstract

**Background and Objective:** There is no consensus regarding modest alcohol consumption in patients with non-alcoholic fatty liver disease (NAFLD) due to conflicting results. The aim of this meta-analysis was to examine the effects of modest alcohol consumption on histological severity, histological course, hepatocellular carcinoma, and long-term clinical outcomes in NAFLD patients.

**Methods:** We searched MEDLINE and EMBASE databases from inception to October 2020 for studies evaluating the effects of modest alcohol consumption among patients with NAFLD. A random-effects meta-analysis using pooled odds ratio (OR) and hazard ratio (HR) was calculated with 95% confidence interval (CI). Study quality was assessed with the Newcastle-Ottawa Scale.

**Results:** Fourteen cross-sectional or cohort studies with aggregate data on 14,435 patients were included in the analysis. Modest alcohol consumption resulted in lower risks for steatohepatitis (OR 0.59; 95% CI 0.45–0.78; *I*^2^ = 12%) and advanced fibrosis (OR 0.59, 95% CI 0.36–0.95; *I*^2^ = 75%). Histological follow-up data showed that modest alcohol use was associated significantly with less steatohepatitis resolution but not with fibrosis progression. The HR for developing hepatocellular carcinoma was 3.77 (95% CI 1.75–8.15; *I*^2^ = 0%). NAFLD patients with modest alcohol intake had a lower mortality risk than lifelong abstainers (HR 0.85; 95% CI 0.75–0.95; *I*^2^ = 64%).

**Conclusion:** This meta-analysis suggests that medical advice for modest alcohol drinking should be made cautiously in caring for an individual patient based on the clinical context. Practically, patients with steatohepatitis or advanced fibrosis should avoid alcohol use, whereas patients with low fibrosis risk may be allowed for modest and safe drinking.

## Introduction

Non-alcoholic fatty liver disease (NAFLD) is the most prevalent chronic liver disorder affecting approximately a quarter of the adult population worldwide (1, 2). NAFLD comprises a continuum of disease severities from steatosis to non-alcoholic steatohepatitis (NASH). It can evolve into an advanced disease that progresses to cirrhosis, liver failure, and an increased risk of hepatocellular carcinoma (HCC) (3). Moreover, NAFLD patients have increased risk of cardiovascular events, other malignancies, and mortality (2, 3). Insulin resistance is a common feature in NAFLD patients, and it is considered the major contributor to the development and progression of the disease (4).

Alcohol consumption in modest quantity is believed to improve insulin resistance, lipid metabolism, and inflammatory status, thereby exerting cardiovascular and metabolic benefits (5). These effects have been shown to reduce the risk of diabetes, cardiovascular disease incidence, and mortality in a J-shape dose-response (6–8). Specific types of alcohols such as red wines and certain drinking patterns, for instance, modest consumption, not binge drinking displayed superior cardiometabolic benefits (9). The benefits of modest alcohol consumption also decrease the risk of developing NAFLD in the general population (10–12). However, recommendations on alcohol consumption among patients with pre-existing NAFLD, where metabolic syndrome and established cardiovascular disease are common comorbidities, remain a topic of vigorous debate, given that the evidence supporting the protective benefits of modest alcohol on liver-related outcomes is less consistent. To date, studies have reported varying results on histological severity, the natural course of liver disease, as well as liver-related outcomes, particularly the development of HCC (13–27). Consequently, there is no current consensus in clinical practice for counseling patients with NAFLD regarding modest alcohol consumption.

Therefore, this systematic review and meta-analysis were performed to comprehensively assess the effects of modest alcohol intake on histological severity, histological progression, and the risk of significant clinical outcomes, namely, the development of cirrhotic complications, HCC, and all-cause death among patients with NAFLD.

## Methods

### Search Trials

Systematic literature review of EMBASE and MEDLINE databases from inception to October 2020 to identify all published studies that evaluated the effects of alcohol consumption on histological severity, histological progression, or clinical events in patients with NAFLD was independently conducted by two investigators (WW and SN). The search strategy that included the terms for “modest alcohol consumption” and “non-alcoholic fatty liver disease” is available in Supplementary Data 1. To ensure the comprehensiveness of eligible studies, the literature review was also conducted from the bibliography of the eligible studies initially retrieved from EMBASE and MEDLINE. This study was conducted according to the Preferred Reporting Items for Systematic Reviews and Meta-Analyses statement (Supplementary Data 2).

### Inclusion and Exclusion Criteria

Eligible studies must be full-text English articles. To evaluate the effects of modest alcohol consumption on histological severity, an eligible study had to be a cross-sectional study of biopsy-proven NAFLD patients and had to report whether modest alcohol intake was associated with NASH or advanced fibrosis compared to abstainers. The pre-requisite outcomes included odds ratio (OR) with 95% confidence interval (CI). For histological progression, eligible cohorts must include serial follow-up liver biopsy to examine how modest alcohol consumption altered the natural history of NAFLD liver histology. These studies must report relative risk (RR), incidence rate ratio (IRR), hazard risk ratio (HR), or standardized incidence ratio (SIR) with 95% CI. To elucidate the effects of modest alcohol on long-term clinical outcomes, eligible studies had to be cohorts reporting RR, IRR, HR, or SIR with 95% CI comparing the risk of the following major clinical events: development of cirrhotic complications (ascites, variceal bleeding, spontaneous bacterial peritonitis, and hepatic encephalopathy), HCC, and all-cause deaths between the two NAFLD cohorts of modest drinkers and abstainers. Modest alcohol drinking was defined as consumption of <21 standard drinks (210 g) per week for men and <14 standard drinks (140 g) per week for women, although some variations were accepted. Two reviewers (WW and SN) independently determined study eligibility. In the first round of screening, titles and abstracts were reviewed to exclude articles that did not fulfill the eligible criteria. The second round of screening involved a full-text review to ensure that the eligible studies fulfilled all inclusion criteria. Disagreements were resolved by discussion with the senior investigator (PC).

### Data Extraction

Extracted data included author, the country where the study was conducted, study design, year of publication, number of participants, recruitment or identification of NAFLD participants, methods used to identify and verify the definition of modest drinkers and abstainers, clinical outcomes, histological classification utilized to diagnose NASH and advanced fibrosis, baseline characteristics of participants, the average duration of follow-up for cohort studies, confounders adjusted in multivariate analysis and adjusted effect estimates with corresponding 95% CI. The appraisal of the quality of the eligible cohort studies was performed according to Newcastle-Ottawa Scale (28). The modified version of this scale was used to appraise cross-sectional studies (29). The quality of each study was evaluated by two investigators (WW and SN), and any differences in opinions were settled by the senior investigator (PC).

### Statistical Analysis

All data analyses were conducted using Review Manager 5.3 software from the Cochrane Collaboration (London, United Kingdom). The generic inverse variance method of DerSimonian and Laird was employed to pool point estimates of all eligible studies, in which the weight of each study for the pooled analysis was in reversal to its standard error (30). Random-effects model was utilized as the eligible studies had different background populations and protocols. The Cochran's Q test and The *I*^2^ statistic were employed to determine statistical heterogeneity. An *I*^2^ value of >75% represented high heterogeneity, 51–75% moderate heterogeneity, 26–50% low heterogeneity, and 0–25% insignificant heterogeneity (31). Publication bias was evaluated with a funnel plot.

## Results

**Figure 1:**
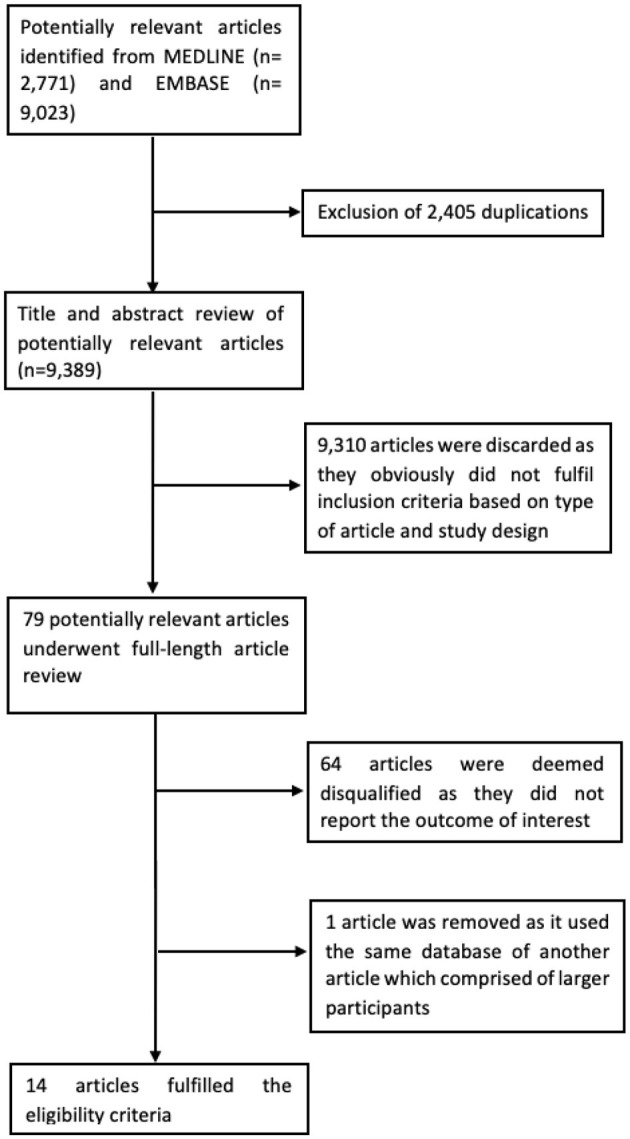
Flowchart of literature review and study selection.

A total of potentially relevant 11,794 articles (9,023 from EMBASE and 2,771 from MEDLINE) were retrieved. After removing 2,405 duplicated articles, 9,389 articles remained for the first-round review. We then excluded 9,310 articles because they did not fulfill the inclusion criteria based on study design and types of articles resulting in 79 remaining articles for the second round full-text review. Fifteen studies fulfilled the inclusion criteria and were included in this study (13–27). However, Younossi et al. (27) and Hajifathalian et al. (25) used the identical database of National Health and Nutrition Examination Survey III (NHANES III); therefore, we selected Hajifathalian et al. due to the larger number of participants. Figure 1 provides an overview of the literature review and study selection process. Tables 1A–C summarizes the study design, characteristics of participants, and Newcastle-Ottawa Scale of the included studies.

**Table 1A:** Characteristics of cross-sectional studies assessing histological severity between non-drinkers and modest drinkers included in the review.

**Author, year**	**Country**	**Participant number(Non-drinker/modest drinker)**	**Age**	**Sex, % men**	**DM, %**	**Definition of modest alcohol drinking**	**Method of assessing alcohol intake**	**Histology scoring system**	**Newcastle Ottawa Scores (selection /comparability /outcome)**
Dixon, 2001	Australia	48/17	41	21	18	<200 g/week	Questionnaire	NAS	3/2/3
Cotrim, 2009	Brazil	57/75	37	31	12	<40 g/day	Interview	Matteoni	4/0/2
Dunn, 2012	USA	252/331	48	34	16	<20 g/day	AUDIT-C, SLDA	NAS	5/2/3
Kwon, 2014	South Korea	25/52	47	44	N/A	<40 g/week	SLDA	NAS	5/2/3
Hagstrom, 2017	Sweden	60/60	56	69	41	<168 g/week	AUDIT-C	NAS, SAF	5/2/3
Ajmera, 2018	USA	117/168	47	30	34	<20 g/day (men) <10 g/day (women)	AUDIT-C	NAS	4/0/3
Kimura, 2018	Japan	208/93	56	45	37	<20 g/day	Questionnaire	NAS, SAF	4/0/3
Mitchell, 2018	Australia	74/91	52	38	41	<210 g/week (men) <140 g/week (women)	Questionnaire	NAS	5/2/3
Yamada, 2018	Japan	101/77	50	52	67.0	<20 g/d	Self-report	NAS	4/0/2
Tan, 2020	Malaysia	55/16	N/A	N/A	N/A	<21 units/week (men) <14 units/week (women)	Self-report	NAS	3/0/3

*AUDIT-C, Alcohol Use Disorder Identification Test–Consumption; DM, diabetes mellitus; NAS, NAFLD Activity Score; N/A, Not available; SAF, Steatosis Activity and Fibrosis; SLDA, Skinner Lifetime Drinking Assessment*.

**Table 1B:** Characteristics of longitudinal follow-up studies on histological progression between non-drinkers and modest drinkers included in the review.

**Author, year**	**Country**	**Participant number**	**Age**	**Sex, % men**	**DM, %**	**Definition of modest alcohol drinking**	**Method of assessing alcohol intake**	**Histology scoring system**	**Follow-up duration (year)**	**Newcastle Ottawa scores (selection/ comparability/ outcome)**
Ekstedt, 2009	Sweden	71	47	72	7	<140 g/week	AUDIT, SLDA	NAS	13.8	4/2/3
Ajmera, 2018	USA	285	47	30	34	<20 g/day (men) <10 g/day (women)	AUDIT	NAS	3.9	4/2/3

*AUDIT-C, Alcohol Use Disorder Identification Test–Consumption; SLDA, Skinner Lifetime Drinking Assessment*.

**Table 1C:** Characteristics of cohort studies comparing the risk of major clinical events between non-drinkers and modest drinkers included in the review.

**Author, year**	**Country**	**Participant number (non-drinker/modest drinker)**	**Age**	**Sex, % men**	**DM, %**	**Definition of modest alcohol drinking**	**Method of assessing alcohol intake**	**Follow-up duration (year)**	**Outcomes**	**Newcastle Ottawa scores (selection/ comparability/ outcome)**
Ascha, 2010	USA	120/68	57	44	73	<30 g/day	Self-report	2.7	HCC	4/0/3
Kimura, 2018	Japan	93/208	56	45	37	<2 units/day	Self-report	6	HCC	4/2/3
Hajifathalian, 2019	USA	3318/1250	49	53	26	<1.5 units/day	Self-report	5.8	Death	4/2/3
Aberg, 2020	Finland	993/6638	54	60	14	<20 g/week	Self-report	11.1	Death	4/1/3

*HCC, Hepatocellular Carcinoma*.

The definition of both abstainers and modest alcohol drinkers varied considerably across included studies from lifetime abstainers to 0.5 standard drink per day and 20 g of alcohol per week to 21 standard drinks (210 g) of alcohol per week, respectively. The definition for maximum amount for modest drinking in men was higher than that of women in three studies (13, 17, 22). All cross-sectional studies were biopsy-based studies. We did not find any studies examining the effects of modest alcohol on the development of cirrhotic complications in the NAFLD population.

### Risk of Steatohepatitis Among NAFLD Patients With Modest Alcohol Consumption

**Figure 2:**
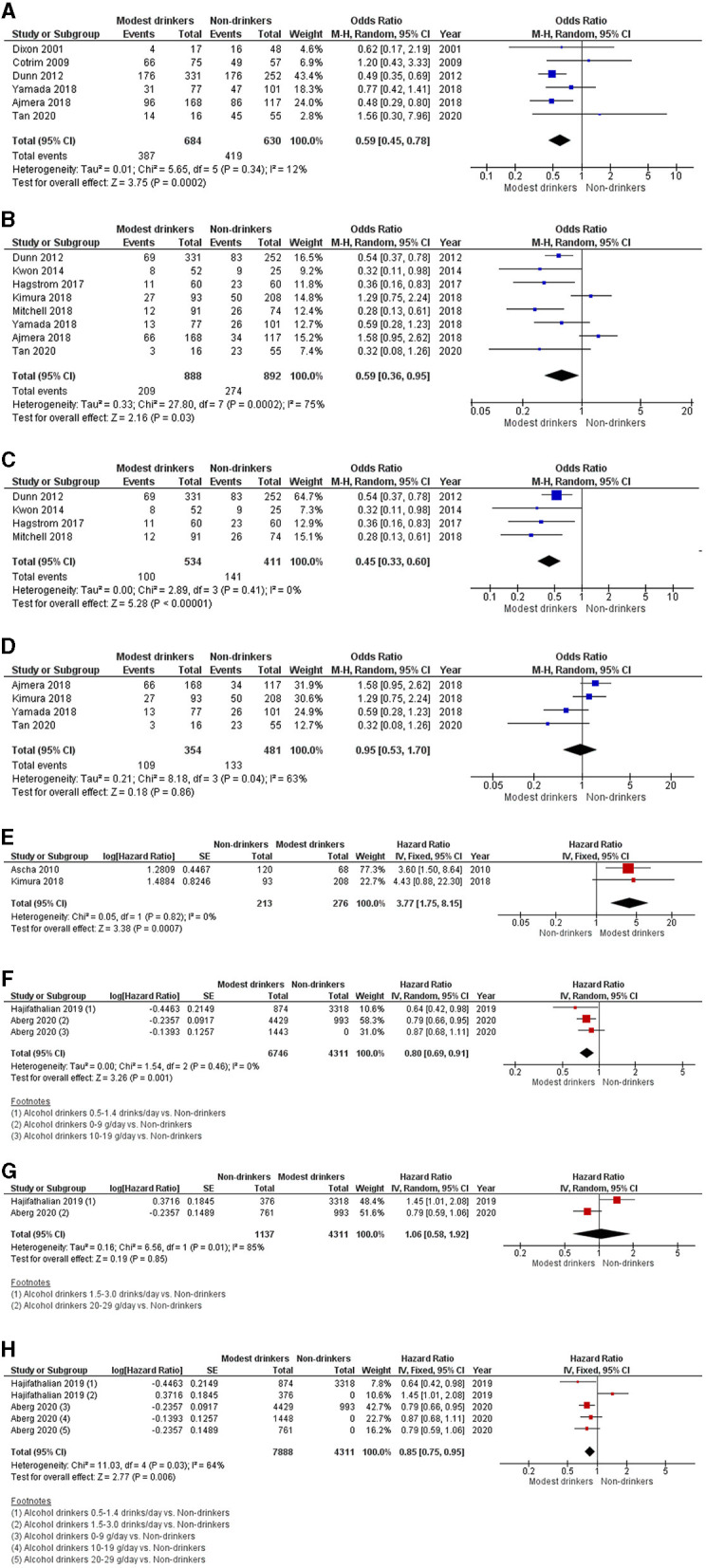
Forest plot of meta-analyses for the association between modest alcohol consumption in NAFLD patients and **(A)** steatohepatitis; **(B)** advanced fibrosis; **(C)** advanced fibrosis among high-quality studies; **(D)** advanced fibrosis among low-quality studies; **(E)** the development of HCC; **(F)** mortality for light alcohol consumption; **(G)** mortality for moderate alcohol consumption; and **(H)** light-to-moderate alcohol consumption.

Six cross-sectional studies investigating the association between modest alcohol drinking and NASH are shown in Figure 2A (13–18). Modest alcohol consumption had a lower prevalence of biopsy-proven NASH among NAFLD patients with a pooled OR of 0.59 (95% CI, 0.45–0.78; *I*^2^ = 12%).

### Risk of Advanced Fibrosis Among NAFLD Patients With Modest Alcohol Intake

Eight cross-sectional studies comparing modest alcohol drinking to non-drinker were identified (13, 16–22). The pooled OR for advanced fibrosis among NAFLD patients with modest alcohol intake was 0.59 (95% CI, 0.36–0.95; *I*^2^ = 75%) (Figure 2B). Additionally, we conducted a sensitivity analysis for advanced fibrosis based on Newcastle-Ottawa scores of those studies. Studies with full Newcastle-Ottawa scores (16, 19, 21, 22) were regarded as high-quality studies and were included in the first sensitivity analysis. Four studies were considered high-quality and yielded a pooled OR of 0.45 (95% CI, 0.33–0.60; *I*^2^ = 0%) (Figure 2C). The other four studies (13, 17, 18, 20) were regarded as low-quality studies and were included in the second sensitivity analysis. Its pooled OR was 0.95 (95% CI, 0.53–1.70; *I*^2^ = 63%) (Figure 2D).

### Systematic Review of the Histopathological Progression of the NAFLD Population With Modest Alcohol Consumption

Two studies investigating the histopathological progression of NAFLD populations were identified and included in the narrative review, but not in quantitative analysis because the results of each study varied significantly and could not be combined (13, 26). Ajmera et al. found that modest alcohol drinking was associated with less NASH resolution among NAFLD patients, with an OR of 0.32 (95% CI, 0.11–0.92) (13). Ekstedt et al. have shown that modest alcohol intake was not significantly associated with risk of a significant fibrosis progression in NAFLD (OR 0.93, 95% CI, 0.10–9.06) (26).

### Risk of HCC Among NAFLD Patients With Modest Alcohol Consumption

The pooled HR from two cohort studies of HCC development was 3.77 (95% CI, 1.75–8.15; *I*^2^ = 0%) (Figure 2E) (20, 23).

### Overall Mortality Among NAFLD Patients With Light, Modest, and Light-To-Modest Alcohol Consumption

The pooled analysis of two cohort studies showed an HR of 0.80 (95% CI, 0.69–0.91; *I*^2^ = 0%) for light consumption (≤ 19 g/day or ≤ 1.4 drink/day), 1.06 (95% CI, 0.58–1.92; *I*^2^ = 85%) for modest consumption (1.5–3.0 drinks/day or 20–29 g/day) and 0.85 (95% CI, 0.75–0.95; *I*^2^ = 64%) for light-to-modest alcohol consumption (≤ 30 g/day) on mortality in NAFLD populations (Figures 2F–H) (24, 25).

### Risk of Publication Bias

**Figure 3:**
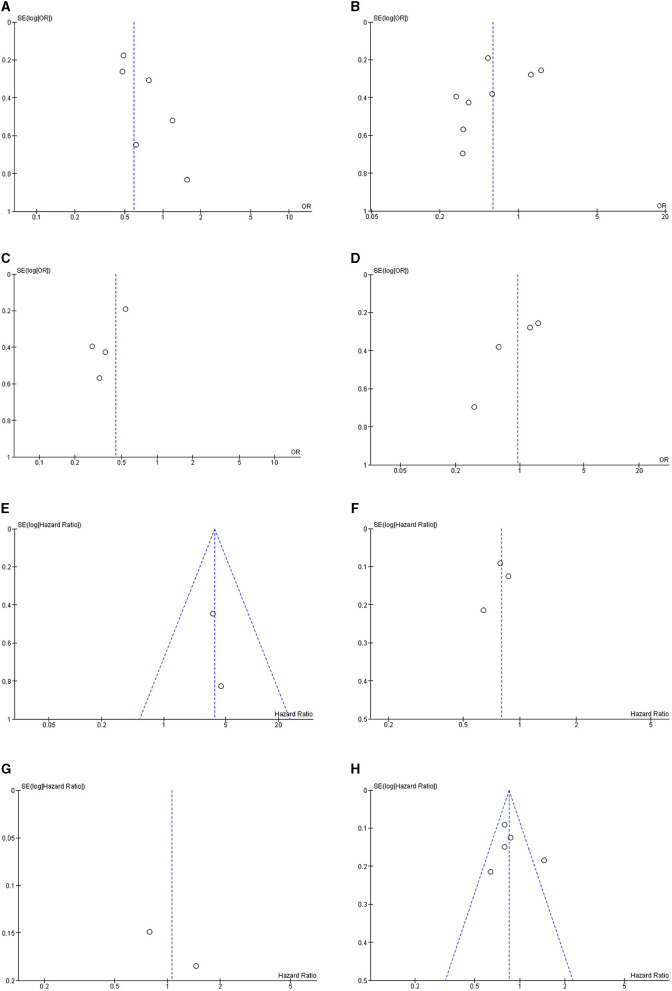
Funnel plot of meta-analyses for the association between modest alcohol consumption in NAFLD patients and **(A)** steatohepatitis; **(B)** advanced fibrosis; **(C)** advanced fibrosis among high-quality studies; **(D)** advanced fibrosis among low-quality studies; **(E)** the development of HCC; **(F)** mortality for light alcohol consumption; **(G)** mortality for moderate alcohol consumption; and **(H)** light-to-moderate alcohol consumption.

Funnel plots of all meta-analyses demonstrated asymmetry and suggested the presence of publication bias (Figures 3A–H).

## Discussion

This comprehensive systematic review and meta-analysis suggest a possible association between modest alcohol consumption and decreased NASH and advanced fibrosis. However, moderate alcohol use may diminish the resolution of NASH and increase risk of HCC in NAFLD patients with advanced fibrosis. In contrast, the data from population-based samples show a protective effect of low-to-moderate alcohol consumption on mortality in patients with NAFLD.

The present meta-analysis observed that modest alcohol drinking was associated with a lower risk of having steatohepatitis and advanced fibrosis in biopsy-proven NAFLD patients. This phenomenon may be explained by the modulation of insulin sensitivity and anti-inflammatory effects of modest alcohol intake, resulting in the attenuation of intrahepatic lipid synthesis, accumulation, and subsequent hepatic steatosis (32–37). The improvement of insulin resistance could moderate lipotoxicity, organelle stress, and hepatocyte injury caused by toxic reactive oxygen species generated by lipid metabolism (32, 38). Interestingly, the effect on advanced fibrosis was only observed in higher-quality studies where ascertainment of alcohol consumption was conducted using validated tools, primarily AUDIT-C and Skinner Lifetime Drinking History. In contrast, the remaining studies involved some degree of self-reported questionnaires or interviews. This could potentially lead to recall bias in the latter group of studies and implies its results since under-reporting may be as high as 40–50% in an alcohol consumption survey and remains a barrier to accurate quantification (39).

The increased risk of HCC among patients with NAFLD who consumed a modest amount of alcohol in our analysis was not unexpected and further emphasized the potentially harmful effects of alcohol. It is known that alcohol is an independent risk factor for the development of HCC both directly via DNA damage from toxic metabolites, oxidative stress, and inflammation and indirectly via chronic liver disease and cirrhosis (20, 40, 41). Furthermore, alcohol in conjunction with diabetes and obesity, as are highly prevalent in the NAFLD study population, also exhibits a synergistic interaction and potentially augments the risk of HCC development (20, 23, 41). Our finding was consistent with a previous study by Kawamura et al. demonstrating that the elevated risk of hepatocarcinogenesis started trending with the daily consumption of 20–39 g of ethanol. However, Kawamura et al. used a light drinker of <20 g per day as the baseline comparator, which differed from our baseline group consisted of abstainers (42). It is also worth noting that all of the patients in Ascha et al., which was weighted at 77% in our analysis, were cirrhotic and were referred for liver transplantation listing due to hepatic decompensation. Therefore, there could be confounding factors for an increased risk of developing HCC via the omission of non-cirrhotic and compensated cirrhosis populations (23). In addition, Kimura et al. found in the multivariate analysis that HCC was associated with fibrosis but not with a mild drinking habit and that all HCC patients had advanced fibrosis (fibrosis stage 3–4) (20). As a result, the interpretation of the risk of HCC development should be made with caution due to the limitations of the NAFLD population. Furthermore, this analysis consisted of only two eligible studies in which HCC was identified exclusively in patients with advanced fibrosis/cirrhosis. Therefore, further research to clarify the actual effect of modest alcohol drinking on the development of HCC across the spectrum of NAFLD patients is needed.

It is well-established that light to moderate alcohol consumption is associated with lower mortality for all-cause, cardiovascular, and cerebrovascular deaths via moderation of metabolic profiles (43, 44). However, studies demonstrating these protective effects were primarily conducted in the general population in national surveys (44). In line with these data, our analyses focused on patients diagnosed with NAFLD and found that modest alcohol consumption was associated with a reduction in all-cause mortality. This outcome could be driven by the decrease as mentioned earlier in the prevalence of advanced fibrosis, which was a significant predictor for long-term overall mortality among biopsy-proven NAFLD patients (45). Consequently, as a knock-on effect of advanced fibrosis reduction, mortality from cirrhosis as the leading cause of death (46) might be attenuated as a result. In addition, cardiovascular death is the leading cause of deaths among NAFLD patients, given the shared atherosclerotic risk factors such as age, diabetes, hypertension, dyslipidemia, insulin resistance, and metabolic syndrome (46, 47). Hence, NAFLD is unsurprisingly considered a risk for cardiovascular disease (48), and it is possible that the cardiometabolic benefits of modest alcohol consumption extended from the general population onto this particular group of NAFLD patients accompanied by atherosclerotic risks. This is particularly evident in Aberg et al. that cardiovascular outcomes, albeit not death-exclusive, were lower among very light drinkers (24). Similarly, we found that all-cause mortality benefits only persisted in light drinkers when patients were grouped according to light or moderate drinking habits. This finding implies that alcohol may not confer its protective effects when consumed beyond a very low threshold. Different types of alcoholic beverages would also need to be accounted for, and further studies to prove this causal relationship in the NAFLD population.

Our meta-analysis has some limitations, which are inherent to the design of the included studies. First, the cross-sectional design of the studies evaluating the effects of alcohol use on the severity of liver disease limits our ability to establish causality of the observed associations. Several studies displayed that moderate alcohol drinkers tended to have higher socioeconomic status, increased physical activity, and less obesity than abstainers (49, 50). These factors have been demonstrated to influence drinking patterns and may affect the severity of liver disease, thereby confounding the association between alcohol use and NAFLD. Second, some of these studies reported incomplete adjustments for potential confounders, and thus reliability of the findings is diminished. Third, although longitudinal cohort studies provided the high quality of the prognostic relevance of modest alcohol use on clinical outcomes in NAFLD, these studies failed to obtain lifetime drinking histories to evaluate past heavy alcohol use. Thus, the population abstaining from alcohol drinking may be enriched for former heavy drinkers, leading to selection bias and more severe liver disease. Fourth, another potential limitation of population-based studies is that NAFLD diagnosis was made using serum biomarkers of steatosis such as fatty liver index and hepatic steatosis index. Accordingly, it is inevitable to have misclassified some of the participants in these studies concerning the presence or absence of NAFLD. Finally, although a random-effects model was applied in this meta-analysis, some findings need to be interpreted cautiously, given the high heterogeneity observed. From the results of the sensitivity analyses, it is assumed that high heterogeneity reflects differences in the tools used for alcohol assessment and characteristics of study populations.

Despite these considerations, this meta-analytic study also has important strengths. First, we believe that the topic of our meta-analysis is clinically relevant, given the conflicting literature on the effects of modest alcohol use in NAFLD and emerging data regarding possible mechanisms of modest alcohol protection for NAFLD. Second, we included studies that performed a liver biopsy to diagnose NASH and assess the liver fibrosis stage, and thus, the histological severity association was ascertained by the gold standard. Third, for the clinical outcomes, the included cohorts had long-term follow-up duration for the pre-specified outcomes to occur adequately. Follow-up time for HCC development, especially in patients with advanced fibrosis and cirrhosis, was as long as six years, while the mean follow-up for mortality was up to 11.1 years. Finally, we used standardized risk estimates from all eligible studies to combine estimates across studies.

## Conclusions

Conflicting results from high heterogeneity of studies and evidence on whether modest alcohol consumption is detrimental or beneficial make clinicians uncertain for counseling abstinence or allowing modest alcohol drinking for potential health benefits. Thus, medical advice should be made cautiously in the context of individual clinical implications. Undoubtedly, patients with NASH and advanced fibrosis should be considered as high-risk groups for progressing to end-stage liver disease; hence, alcohol drinking should be avoided. On the contrary, NAFLD patients with low fibrosis risk may be allowed for modest and safe drinking. Thus, there is an urgent need to clarify possible variable impacts of modest alcohol use across the different stages of NAFLD.

## Data Availability Statement

The raw data supporting the conclusions of this article will be made available by the authors, without undue reservation.

## Author Contributions

WW, SN, and PC were involved in the study design, data search and collection, and writing of the manuscript. PC performed statistical analysis. All authors approved the final version of the manuscript.

## Conflict of Interest

The authors declare that the research was conducted in the absence of any commercial or financial relationships that could be construed as a potential conflict of interest.

## Publisher's Note

All claims expressed in this article are solely those of the authors and do not necessarily represent those of their affiliated organizations, or those of the publisher, the editors and the reviewers. Any product that may be evaluated in this article, or claim that may be made by its manufacturer, is not guaranteed or endorsed by the publisher.

## Abbreviations

NAFLD: non-alcoholic fatty liver disease
NASH: non-alcoholic steatohepatitis
HCC: hepatocellular carcinoma
OR: odds ratio
HR: hazard ratio
CI: confidence interval
RR: relative risk
IRR: incidence rate ratio
HR: hazard risk ratio
SIR: standardized incidence ratio
NHANES III: National Health and Nutrition Examination Survey III
AUDIT-C: Alcohol Use Disorder Identification Test–Consumption
DM: diabetes mellitus
NAS: NAFLD Activity Score
N/A: Not available
SAF: Steatosis Activity and Fibrosis
SLDA: Skinner Lifetime Drinking Assessment.
