# Supplementary material for: The Effects of Modest Alcohol Consumption on Non-alcoholic Fatty Liver Disease: A Systematic Review and Meta-Analysis
Source: Front Med (Lausanne). 2021 Aug 27;8:744713. doi: 10.3389/fmed.2021.744713 (PMC8429798; doi:10.3389/fmed.2021.744713)
Supplement: Supplementary file 1 [file Table_1.DOCX]

Supplementary Data 1

Search strategy

**EMBASE**

1. 'nonalcoholic fatty liver'/exp OR 'nonalcoholic fatty liver'
2. 'nonalcoholic fatty liver disease'/exp OR 'nonalcoholic fatty liver disease'
3. ‘nonalcoholic steatohepatitis’/exp OR ‘nonalcoholic steatohepatitis’
4. 'NASH'/exp OR 'NASH'
5. 'NAFLD'/exp OR ‘NAFLD'
6. ‘NAFL’/exp OR ‘NAFL’
7. #1 OR #2 OR #3 OR #4 OR #5 OR #6
8. 'ethanol'/exp OR ‘ethanol’
9. ‘alcohol'/exp OR 'alcohol'
10. ‘alcohol drinking'/exp OR 'alcohol drinking'
11. ‘alcohol consumption'/exp OR 'alcohol consumption'
12. ‘alcoholic beverages'/exp OR ‘alcoholic beverages'
13. #8 OR #9 OR #10 OR #11 OR #12
14. #7 AND #13

**MEDLINE**

1. nonalcoholic fatty liver.mp. or exp non-alcoholic fatty liver/
2. non-alcoholic fatty liver.mp. or exp non-alcoholic fatty liver/
3. nonalcoholic steatohepatitis.mp. or exp nonalcoholic steatohepatitis/
4. non-alcoholic steatohepatitis.mp. or exp non-alcoholic steatohepatitis/
5. nonalcoholic liver disease.mp. or exp nonalcoholic liver disease/
6. non-alcoholic liver disease.mp. or exp non-alcoholic liver disease/
7. NAFLD.mp. or exp NAFLD/
8. NASH.mp. or exp NASH/
9. NAFL.mp. or exp NAFL/
10. 1 or 2 or 3 or 4 or 5 or 6 or 7 or 8 or 9
11. alcohol.mp. or exp alcohol/
12. ethanol.mp. or exp ethanol
13. alcohol drinking.mp. or exp alcohol drinking/
14. alcohol consumption.mp. or exp alcohol consumption/
15. alcoholic beverages.mp. or exp alcoholic beverages/
16. 11 or 12 or 13 or 14 or 15
17. 10 and 16
